# Thirty years of research on traumatic brain injury rehabilitation: a bibliometric study

**DOI:** 10.3389/fneur.2023.1170731

**Published:** 2023-05-15

**Authors:** Yang Liu, Xiaomeng Yao, Jinghua Qian

**Affiliations:** ^1^School of Sports Medicine and Rehabilitation, Beijing Sport University, Beijing, China; ^2^Viterbi School of Engineering, University of Southern California, Los Angeles, CA, United States

**Keywords:** Traumatic Brain Injury, TBI, Rehabilitation, bibliometric, network, hotspots

## Abstract

**Background:**

Traumatic brain injury (TBI) is a major public health concern with far-reaching consequences on individuals’ lives. Despite the abundance of works published on TBI rehabilitation, few studies have bibliometrically analyzed the published TBI rehabilitation research. This study aims to characterize current international trends and global productivity by analyzing articles on TBI rehabilitation using bibliometric approaches and visualization methods.

**Methods:**

We conducted a bibliometric analysis of data retrieved and extracted from the Web of Science Core Collection database to examine the evolution and thematic trends in TBI rehabilitation research up until December 31, 2022. The specific characteristics of the research articles on TBI rehabilitation were evaluated, such as publication year, countries/regions, institutions, authors, journals, research fields, references, and keywords.

**Results:**

Our analysis identified 5,541 research articles on TBI rehabilitation and observed a progressive increase in publications and citations over the years. The United States (US, 2,833, 51.13%), Australia (727, 13.12%), and Canada (525, 9.47%) were the most prolific countries/regions. The University of Washington (226, 4.08%) and Hammond FM (114, 2.06%) were the most productive institution and author, respectively. The top three productive journals were *Brain Injury* (862; 15.56%), *Archives of Physical Medicine and Rehabilitation* (630; 11.37%), and *Journal of Head Trauma Rehabilitation* (405, 7.31%). The most frequent research fields were Rehabilitation, Neurosciences, and Clinical Neurology. Co-citation references primarily addressed “outcome assessment,” “community integration” and “TBI management,” and “injury chronicity” and “sequelae” have gained more attention in recent years. “Mild TBI,” “outcome,” “stroke” and “children” were the commonly used keywords. Additionally, the analysis unveiled emerging research frontiers, including “return to work,” “disorder of consciousness,” “veterans,” “mild TBI,” “pediatric,” “executive function” and “acquired brain injury.”

**Conclusion:**

This study provides valuable insights into the current state of TBI rehabilitation research, which has experienced a rapid increase in attention and exponential growth in publications and citations in the last three decades. TBI rehabilitation research is characterized by its multi-disciplinary approach, involving fields such as Rehabilitation, Neurosciences, and Clinical Neurology. The analysis revealed emerging research subjects that could inform future research directions.

## 1. Introduction

Traumatic brain injury (TBI) constitutes a significant global public health and socioeconomic issue, with far-reaching consequences on patients’ physical, cognitive, psychological, social, emotional, and behavioral well-being. TBI is a leading cause of death and disability in young adults (1), with an annual incidence proportion of 295 per 100,000 of all ages (2). Among more than 69 million new cases of TBI diagnosed globally each year (3), over 55.5 million cases result in disability (2). Common injury mechanisms of TBI include unintentional falls (4), automobile accidents, firearm-related injury (5, 6), sports, and assault. With the increase in the elderly population in developed countries, TBI due to unintentional falls (secondary) could become an increasing public health and socioeconomic issue. Between 2006 and 2014, the age-adjusted rates of TBI-related emergency department (ED) visits rose by almost 54%, from 521.6 to 801.9 per 100,000 population, while the death rate decreased by 6% (7). Faster transport to trauma centers and more effective treatment may account for the decline in mortality, which has led to an increase in the number of TBI survivors who require rehabilitation and related care (8). The overall goal of rehabilitation following TBI is to assist the person in achieving the highest degree of cognitive, functional, and physical capacity to maximize an independent post-injury life (9). Given the significant impact of TBI on individuals and society, understanding the current state of TBI rehabilitation research is crucial.

Bibliometrics refers to the statistical analysis of bibliographic data from scientific publications, such as articles and books, which is utilized to evaluate and reveal the structure of research and its productivity and trends (10). This analysis facilitates the examination of thousands of publications within a specific subject or research field, enabling the identification of the most influential publications, as well as collaboration among countries, authors, institutions, and active journals (11). To our knowledge, only one bibliometric study has been published explicitly concentrating on TBI rehabilitation. However, this study had limitations in terms of bibliometric methods and the time frame examined. Mojgani et al. (8) developed a Python script to analyze relevant articles on TBI rehabilitation as of 2017 and found that mild TBI and concussion were highly discussed hot topics. While the earlier publication offered insights into facets of TBI rehabilitation, a more comprehensive bibliometrics analysis could potentially enrich the field by elucidating research themes that have garnered attention in the past decades. Recently, the fusion of visualization and data mining techniques has bolstered bibliometric approaches, but these novel methods have not yet been applied to studies examining TBI rehabilitation.

This study aims to provide a comprehensive assessment and a clear understanding of the scientific articles on TBI rehabilitation up until December 31, 2022, through state-of-the-art bibliometric approaches. The primary objective is to understand the patterns of TBI rehabilitation studies from multiple perspectives, including the temporal evolution of scientific publications, geographic distribution, lead authors and journals, research fields, and current trends.

## 2. Methods

### 2.1. Search strategy

We conducted a systematic search for English articles from the Web of Science Core Collection (WoSCC) database using the medical subject headings and topic terms: “Traumatic Brain Injury” and “Rehabilitation.” Our search strategy was as follows: TS = (Traumatic Brain Injury) AND TS = (Rehabilitation) AND Document Type = (Article) AND Language = (English), with a Publication Date set from 1970 to 2022. To minimize potential bias from frequent database updates, we retrieved literature up to December 31, 2022.

### 2.2. Data collection

Two researchers (YL and XY) independently retrieved the literature, extracted the data, and cross-checked their results. Any discrepancies were resolved through discussion or consultation with the senior author (JQ). The data of the included articles from WoSCC were downloaded using the “Custom selection” option, which selected all 29 fields for custom export in “plain text file” format (12). Missing data was manually completed based on the original literature data.

### 2.3. Statistical analysis

Co-Occurrence (COOC, version 13.4) (13) is a bibliometrics and knowledge graph visualization software. To prepare the data for analysis, COOC was used to merge the downloaded txt format files, eliminate duplicates, convert files to an xlsx format file, merge synonyms of keywords, and clean the data. VOSviewer (1.6.19), a visualizing bibliometric networks software (14), was used to analyze cooperation among countries, institutions, and authors, as well as author co-citation and keyword co-occurrence. CiteSpace (6.1.R6 64-bit Advanced), a visual analytic computer program developed by Dr. Chen Chaomei in Java, was used for the reference co-citation analysis and mapping of visualization knowledge domains (15, 16). We extracted the academic journal information, including impact factors and category rank, from the Journal Citation Reports^™^ (JCR) of 2021. Statistical analysis was conducted using Microsoft Excel 2022 (Microsoft, Redmond, Washington, United States). Datawrapper, a web-based tool (17), was used to draw the world choropleth map and institution symbol map. The threshold for entering the next stage of publication was set at publishing more than 100 articles per year.

We used the country/region, author, reference, and keyword as the node to draw the corresponding network maps for visual analysis, and the cluster labels for reference analysis that reveal the main topics were extracted from the title word lists using log-likelihood ratio (18). The modularity value and silhouette score are usually used to assess the clusters. If the modularity value is over 0.3 and silhouette score is over 0.7, it means the cluster community structure is significant and the members have high homogeneity (15, 19), indicating the clustering result is meaningful and efficient (20). In these network maps, node size is positively correlated with the frequency of occurrence, while the connecting lines indicate co-occurrence between two nodes, and the thickness of the lines indicates the strength of the co-occurrence. The cluster timeline map permits the clearly identification of different research trends and their evolution (18).

## 3. Results

### 3.1. Study selection and data processing

**Figure 1:**
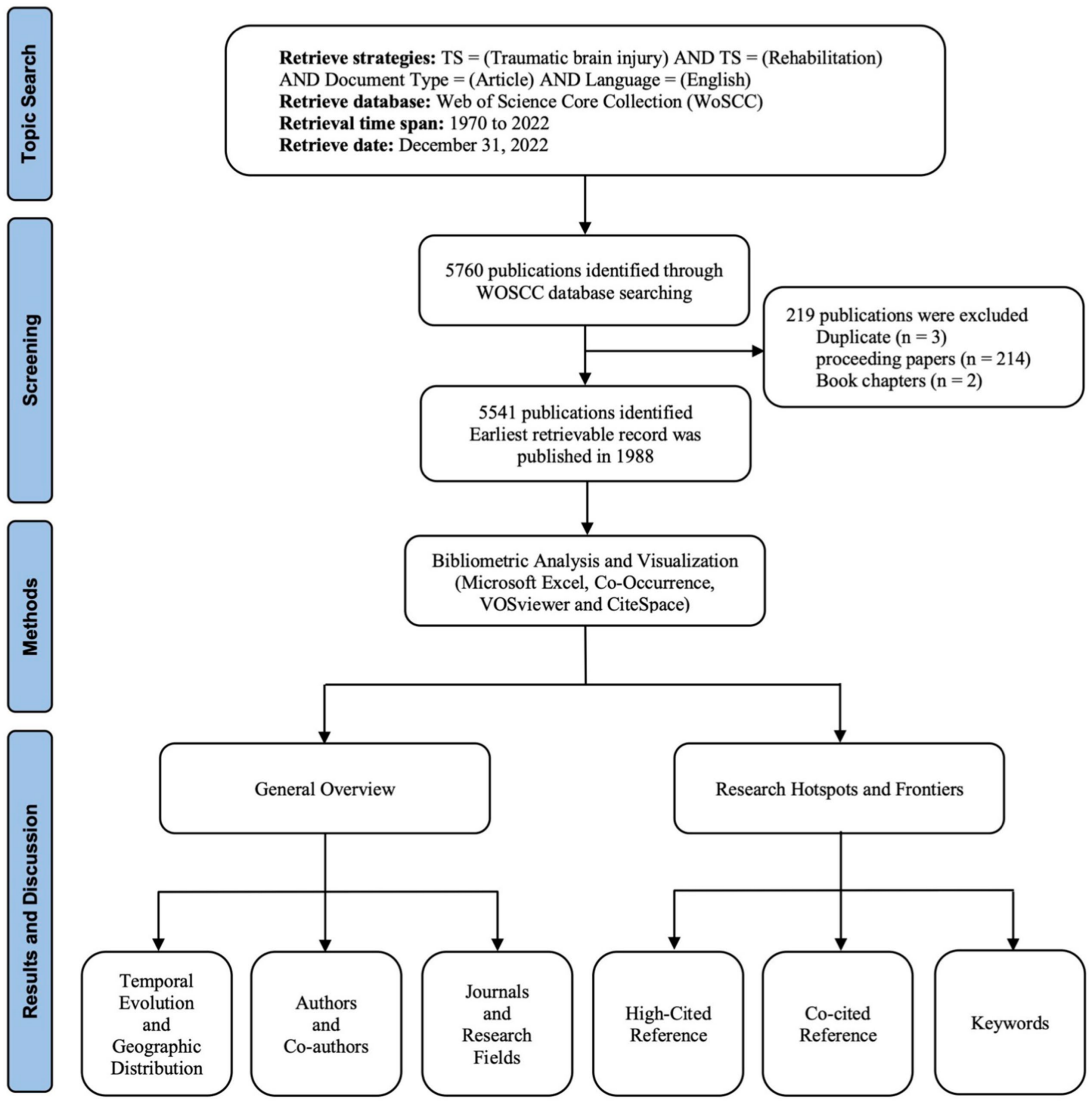
Flowchart of literature screening included in this study.

According to our search strategy, the earliest retrievable record from WOSCC was published in 1988. Thus, this study period spanned from 1988 to the end of 2022. Our search strategy yielded a total of 5,760 articles after three duplicates were excluded. We also eliminated 214 proceeding papers and two book chapters, leaving us with a total of 5,541 studies for analysis (Figure 1).

There were 1,017 missing fields of the author keywords, and 65 keywords were still missing after copied the supplementary keywords to the author keywords. Consequently, the 65 pieces of literature were excluded from the keyword analysis. We utilized COOC to merge 1,147 synonyms for the identified keywords. After we merged synonyms and removed meaningless words, 6,953 keywords appeared in 5,541 studies. Additionally, we observed 49 missing fields for institutions and countries/regions, which we manually completed based on the original literature.

### 3.2. Analysis of annual global publications output

**Figure 2:**
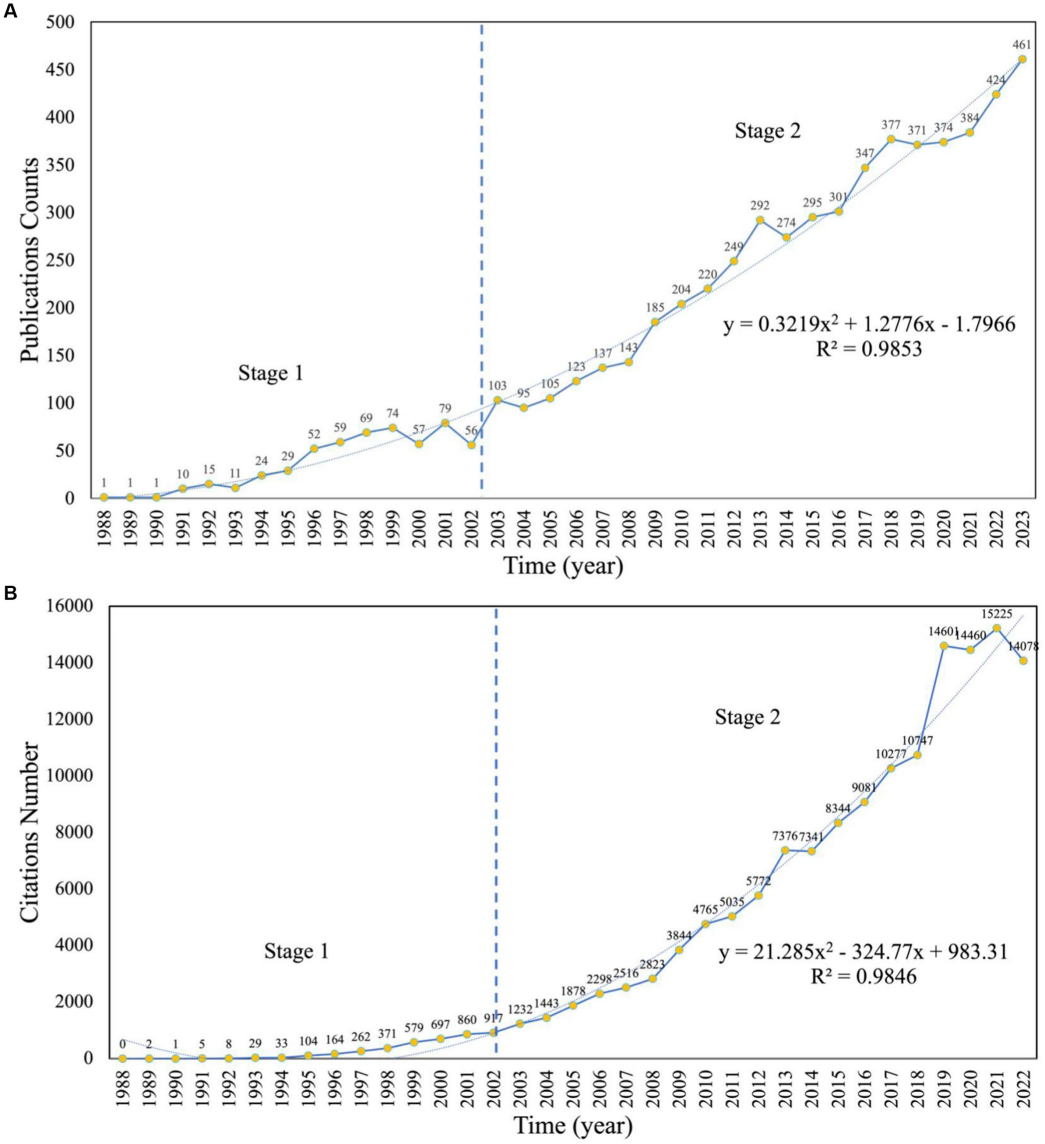
The publications counts **(A)** and the citations number **(B)** per year on TBI rehabilitation research between 1988 and 2022.

We conducted an analysis of the temporal evolution of publications in TBI rehabilitation research from 1988 to 2022. The output of annual publications on TBI rehabilitation is shown in Figure 2A, which reveals two stages: stage 1 (1988–2002) and stage 2 (2003–2022). During the 15-year period of stage 1, the number of publications grew slowly, with an average of 35.87 publications per year. In stage 2, which spanned 20 years after 2002, there was an average of 250.15 articles published per year. The number of annual global publications increased from 56 in 2002 to 424 in 2022, reflecting a growth of 657.1%. The output exceeded 300 between 2017 and 2022 and peaked at 424 in 2022. The fitted curve (*R*^2^ = 0.9853) shows that the number of publications increases exponentially, with the regression analysis indicating that 461 articles will be published in 2023.

Figure 2B displays the number of citations obtained from WOSCC’s citation report, which also fluctuates and increases year by year. A total of 147,831 citations have been cited, with an average of 26.67 citations per article. This increase has been particularly heightened after 2002 and is reflected in the fitted curve (*R*^2^ = 0.9846), indicating an exponentially increasing trend that coincides with the analysis of published articles.

### 3.3. Analysis of geographic distribution

**Figure 3:**
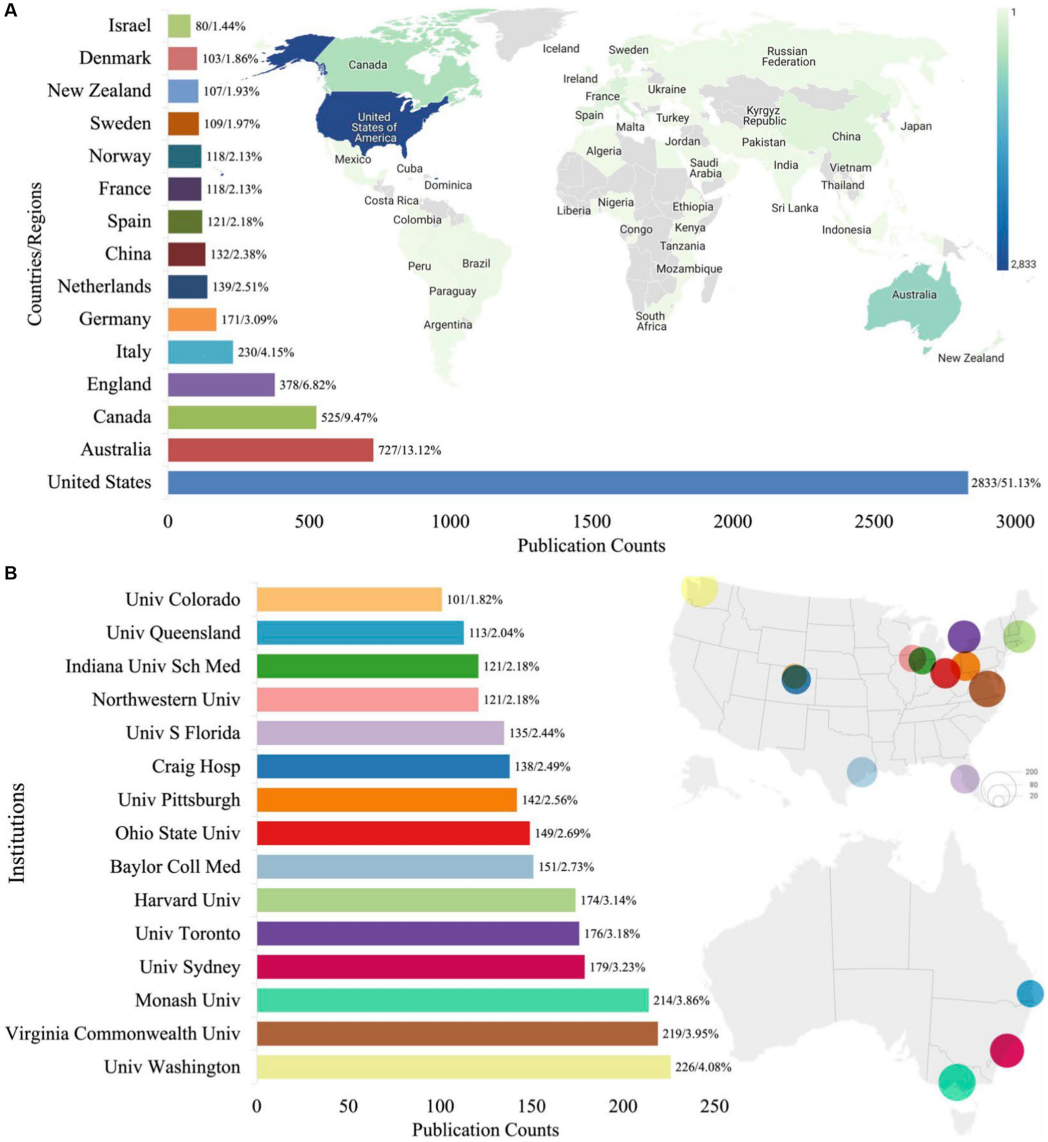
The distribution of the top productive world countries/regions **(A)** and institutions **(B)** on TBI rehabilitation (Univ: University; Sch: School; S: South; Hosp: Hospital; Coll: College; MED: Medicine).

TBI rehabilitation had 101 countries/regions and 5,147 institutions publishing at least one article in this field from 1988 to 2022. Figure 3 illustrates the top 15 countries/regions and institutions with the highest number of published articles. The US was a dominant contributor to collaborative TBI rehabilitation research, with 2,833 articles published, representing 51.13% of the total articles. Australia and Canada also made significant contributions to TBI rehabilitation research, with a total of 1,252 articles (accounting for 22.59% of all publications). This finding underscores the potential for co-research collaborations to support other developing countries/regions. Additionally, an analysis of the collaborations between countries/regions revealed that regional clusters were generally formed based on geographical location.

The University of Washington made the greatest contribution to TBI rehabilitation research and participated in publishing 226 articles, accounting for 4.08% of the total. The top 15 institutions are all located in the US, Canada, and Australia. Collaboration network maps of the countries/regions and institutions were plotted to provide a visual representation of the cooperative relationships involved in TBI rehabilitation research (Supplementary Figure S1). The maps show the 30 countries/regions (Supplementary Figure S1A) and 149 institutions (Supplementary Figure S1B) that published 20 or more articles during the same period. Notably, the US is the leading country in this field, with close collaborative relationships observed between the US, Canada, and Australia. Moreover, the dense lines in the collaboration network maps suggest a high level of cooperation among the institutions involved in this field.

### 3.4. Analysis of authorship

During the period from 1988 to 2022, a total of 17,410 authors participated in TBI rehabilitation studies. The top 10 most productive authors are displayed in Table 1, with Hammond FM from Indiana University School of Medicine ranking first with 114 articles and having the highest total link strength. To further examine the collaborations between authors in this field, we constructed a co-occurrence network map (Supplementary Figure S2A) of the 78 authors who published at least 20 articles. The abundance of interconnections between researchers indicates prevalent cooperation within the field.

**Table 1:** Top 10 most productive authors in TBI rehabilitation research between 1988 and 2022.

Author name	Nationality	Institution	*n*	%	TLS
Hammond FM	US	Indiana University School of Medicine	114	2.06	333
Ponsford J	Australia	Monash University	102	1.84	96
Sherer M	US	TIRR Memorial Hermann	84	1.52	218
Corrigan JD	US	Ohio State University	71	1.28	166
Fleming J	Australia	University of Queensland	69	1.25	42
Sander AM	US	Baylor College of Medicine	66	1.19	153
Whyte J	US	Moss Rehabilitation Research Institute	65	1.17	134
Nakase-Richardson R	US	University of South Florida	62	1.12	137
Arango-Lasprilla JC	Spain	Basque Foundation for Science	61	1.10	111
Hart T	US	Moss Rehabilitation Research Institute	59	1.06	189

US, United States; TLS, Total Link Strength.

Author co-citation is a significant indicator of an author’s influence within a specific field. Supplementary Figure S2B depicted a co-citation network map of corresponding authors, which includes 73 authors who were co-cited at least 200 times. Node size corresponds to their co-citation counts, while lines indicate their co-citation relationships. The top ten most co-cited authors include Corrigan JD (co-citations *n* = 1,121), Cicerone KD (875), Ponsford J (850), Levin HS (834), Prigatano GP (749), Teasdale G (705), Kreutzer JS (684), Sherer M (678), Wechsler D (616), and Malec JF (609). Five of these authors are also among the top 10 most productive authors.

### 3.5. Analysis of journals and research fields

From 1988 to 2022, 697 journals published at least one article on TBI rehabilitation. The top 10 most productive journals have been identified, accounting for about 51.87% of the total publication. As shown in Table 2, *Brain Injury* published the largest number of articles, accounting for 15.56% of the total. *Archives Of Physical Medicine And Rehabilitation* (Ranked second, published 630 articles, accounting for 11.37%) has maintained a leading position in the research field of rehabilitation and sports science for many years in a row (Q1, Q2), with the highest total citations. We also obtained the top 10 journals quality updates according to the JCR of 2021 (Table 3). Among the top 10 productive journals, six journals ranked in the second quarter or higher in the relevant research category.

**Table 2:** Top 10 most productive journals and their newest impact fact.

Journal name	*n*	%	IF	NC	AC
*Brain Injury*	862	15.56	2.167	23,831	27.65
*Archives of Physical Medicine and Rehabilitation*	630	11.37	4.060	27,617	43.84
*Journal of Head Trauma Rehabilitation*	405	7.31	3.117	9,588	23.67
*Neuropsychological Rehabilitation*	238	4.30	2.928	5,129	21.55
*Disability and Rehabilitation*	193	3.48	2.439	3,360	17.41
*Journal of Neurotrauma*	159	2.87	4.869	6,434	40.47
*Neurorehabilitation*	139	2.51	1.986	1,599	11.5
*American Journal of Physical Medicine and Rehabilitation*	87	1.57	3.959	2,887	33.18
*Journal of Rehabilitation Medicine*	85	1.53	3.412	2,258	26.56
*Journal of Rehabilitation Research and Development*	76	1.37	1.277	4,133	54.38

IF, Impact factor (2021); NC, Number of citations; AC, Average citation per article.

**Table 3:** JCR categories and journal quality information of the top 10 most productive journal.

JCR category	Category quartile	Category rank
Neurosciences, Rehabilitation	Q4, Q3	237/275, 38/68
Rehabilitation, Sport Sciences	Q1, Q1	11/68, 22/88
Clinical Neurology, Rehabilitation	Q3, Q2	123/212, 19/68
Neurosciences, Psychology	Q3, Q3	199/275, 41/80
Rehabilitation	Q2	28/68
Clinical Neurology, Critical Care Medicine, Neurosciences	Q2, Q2, Q2	60/212, 12/35, 95/275
Clinical Neurology, Rehabilitation	Q4, Q3	178/212, 41/68
Rehabilitation, Sport Sciences	Q1, Q2	13/68, 25/88
Rehabilitation, Sport Sciences	Q1, Q2	16/68, 35/88
Rehabilitation	Q3	37/65

JCR, Journal Citation Reports.

**Figure 4:**
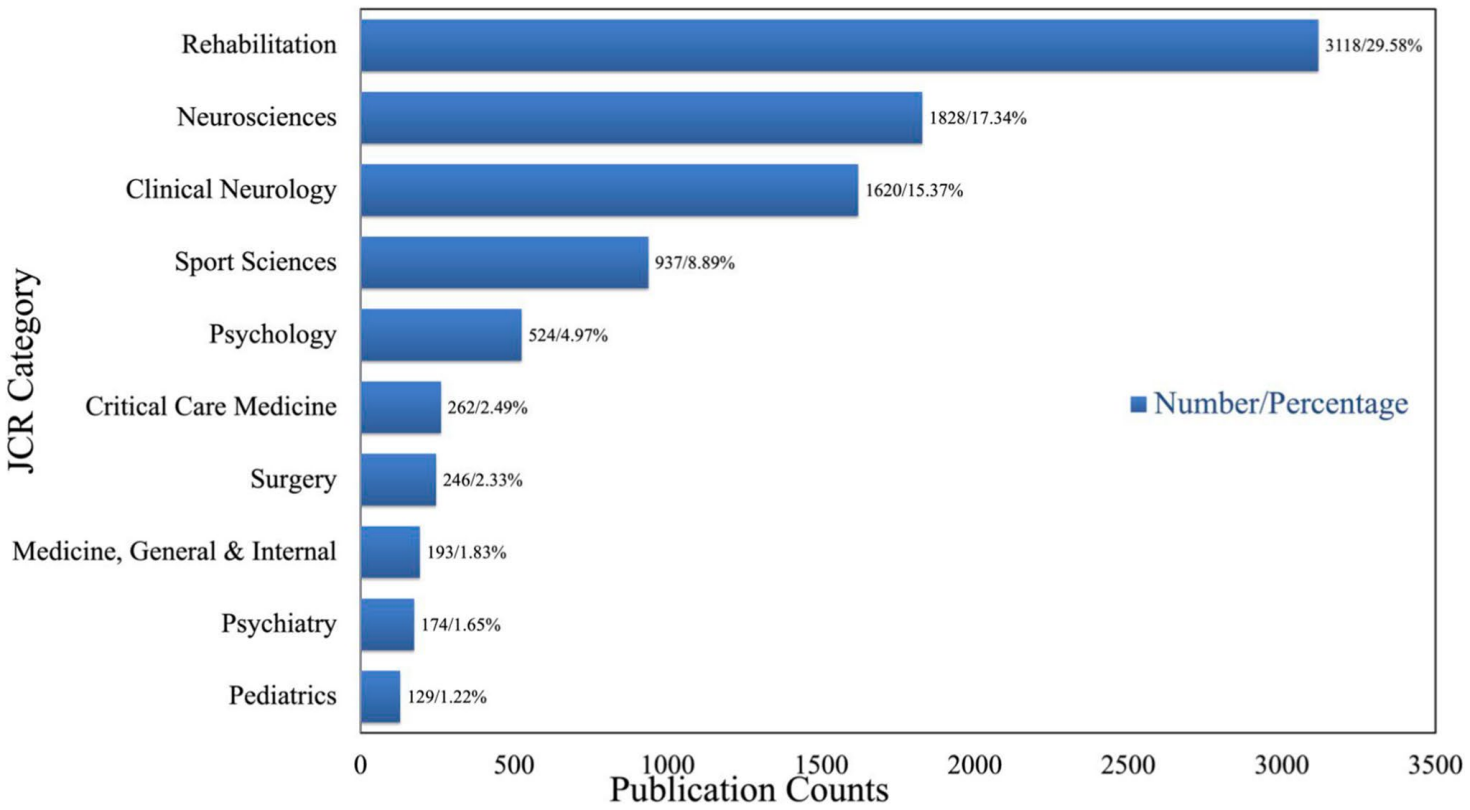
Publication counts based on research fields in TBI rehabilitation research.

Furthermore, we conducted an analysis of the research fields involved in TBI rehabilitation and identified 117 research fields. Among these, we determined the top 10 research fields, which collectively accounted for 85.67% of total publications (Figure 4). Rehabilitation was found to be the most prevalent research field with 3,118 publication counts, representing 29.58% of all publications. Following closely behind were Neurosciences (*n* = 1,828, 17.34%) and Clinical Neurology (*n* = 1,620, 15.37%). Collectively, these top 3 research fields accounted for approximately 62.28% of total publications.

### 3.6. Analysis of cited references

The most cited references are generally considered to have the greatest impact on the scientific community (21). We have listed the top 10 most cited articles in Supplementary Table S1. The studies “Treatment of traumatic brain injury with moderate hypothermia (22)” and “Position Statement: Definition of Traumatic Brain Injury (23)” rank first and second, respectively, with 939 and 826 citation times. The former is the oldest of the top 10 most cited articles, indicating its influential position among these articles. The articles “Global, regional, and national burden of neurological disorders, 1990–2016: a systematic analysis for the Global Burden of Disease Study 2016 (2)” and “Constraint-Induced Movement Therapy: A new family of techniques with broad application to physical rehabilitation—A clinical review (24)” rank third and fourth, with citation times exceeding 600 (693 and 608, respectively).

### 3.7. Analysis of reference co-citation

Reference co-citation analysis is a method used to identify popular research topics (25). In the present study, we examined 115,437 publications that were cited in the reference sections of the analyzed articles. Using these data, we generated a density web map that includes 194 highly cited publications, each of which was cited at least 50 times (Supplementary Figure S3). Among these publications, the most frequently co-cited studies in the reference sections were those conducted by Teasdale G (1974) (26), Rappaport M (1982) (27), Jennett B (1975) (28), Wilson JTL (1998) (29), and Levin HS (1979) (30).

By utilizing the clustering feature of CiteSpace software, we were able to identify seven major research topic clusters. The modularity value and the mean silhouette score were 0.8913 (>0.3) and 0.9483 (>0.5), respectively. As depicted in Supplementary Figure S4A, each node represents a cited reference, the node size corresponds to its citation frequency, and the lines illustrate the co-citation relationships. The largest cluster (#0) is “TBI,” followed by “post-acute care” (#1), “biologics effectiveness research” (#2), “sequelae” (#3), “veteran population” (#4), “injury chronicity” (#8), and “sequelae” (#10). The more significant a cluster is, the more interest it receives from researchers. Furthermore, the timeline view of reference co-citation highlights that “injury chronicity” and “sequelae” are two emerging research topics in TBI rehabilitation (Supplementary Figure S4B).

### 3.8. Analysis of keywords co-occurrence

Keywords are critical to reflect the main information in a research article (31), and help identify research hotspots and anticipate future directions (32). We portrayed a keyword co-occurrence network map, with 71 keywords that appeared at least 50 times in TBI rehabilitation research articles (Supplementary Figure S5). We compiled a list of the top 20 keywords that appeared most frequently in TBI rehabilitation studies (Table 4). “TBI” occurred 2,279 times, making it the most frequent keyword, followed by “Rehabilitation” and “Brain injuries.”

**Table 4:** Top 20 most occurrences keywords in TBI rehabilitation research.

Keywords	*n*	Keywords	*n*
Traumatic brain injury (TBI)	2,279	Cognitive rehabilitation	180
Rehabilitation	1914	Recovery	179
Brain injuries	1,218	Spinal cord injury	168
Mild traumatic brain injury	353	Traumatic	162
Outcome	322	Veterans	147
Stroke	275	Disability	138
Depression	199	Acquired brain injury	124
Children	195	Memory	114
Quality of life	190	Pediatric	113
Cognition	182	Outcome assessment	105

The different colors of the nodes in Supplementary Figure S5 represent the average publication year of the articles in which the keyword appeared, with yellow indicating more recent publications (33). Emerging research hotspots are suggested by the keywords in more recent publications, such as “return to work (average publication year: 2017.68),” “disorder of consciousness (2017.62),” “veterans (2016.86),” “mild TBI (2016.63),” “pediatric (2016.55),” executive function (2016.42),” and “acquired brain injury (2016.29).”

## 4. Discussion

### 4.1. General overview of the TBI rehabilitation research

This is the first bibliometric study using visualization tools to analyze the global research trends in TBI rehabilitation. The increasing number of publications and citations in this field suggests increased attention to TBI rehabilitation research over the years. Since 2002, interest in TBI rehabilitation had soared, leading to the publication of 350 articles annually in the past 5 years, with projections of over 460 articles per year in the coming years.

Collaboration is a key characteristic of TBI rehabilitation research, of which growing development led by increasing cross-country and cross-institutional cooperation serves as convincing evidence. The US, Australia, and Canada have published the most literature on TBI rehabilitation and have also ranked in the top five countries for research on spinal cord injury rehabilitation (34) and sport-related TBI rehabilitation (35). The most prolific and influential authors in the field have been identified, providing valuable collaboration and consultant information for researchers worldwide. The prevalent interconnectivity among researchers suggests that collaboration is common, aligning with prior research that co-authorship is associated with higher citation rates, while single-author papers accounted for only 8% of rehabilitation research documents (8).

TBI rehabilitation research has been recognized and disseminated by top journals of rehabilitation and sports science. Our analysis also highlights the importance of multidisciplinary approaches to TBI rehabilitation, as demonstrated by the involvement of fields such as Rehabilitation, Neurosciences, and Clinical Neurology. It is a fast-growing field that has become more active than it was decades ago (36). Additionally, sport-related TBI rehabilitation is an indispensable subset of TBI rehabilitation, and recent bibliometric studies have examined its impact over the past 20 years (35).

### 4.2. Research hotspots and frontiers of TBI rehabilitation research

The analysis of cited references revealed the most impactful articles, forming the foundation of knowledge for future studies in this field. The 10 most cited articles were published on average nearly 15 years before this study, suggesting that the knowledge in the field does not become obsolete quickly and can affect papers published years later (8). Based on the highly cited publications identified through co-citation analysis, it is suggested that “outcome assessment” was a vital research topic in this field.

The analysis of reference co-citations identified the seven major clusters that primarily focus on outcome assessment (37–39), community integration (40, 41), and TBI management (42–45). For outcome assessment, the keywords “outcome” and “quality of life” are commonly occurring in this field. However, unlike process measures, which are easily described, outcomes and quality of life can often be surprisingly difficult to define and measure (46). Various outcome assessment scales including the Glasgow Outcome Scale with or without extended scores (28), Disability Rating Scale (39, 47), Functional Independence Measure (38, 48), Community Integration Questionnaire (CIQ) (40), and the Quality of Life after Brain Injury (49), have been proposed and used to assess disability after TBI. Their continued use in research highlights their importance and relevance.

Community integration (CI) is an essential aspect of rehabilitation for individuals with TBI, encompassing home integration, social integration, and productive activities (50). It is not just about physical recovery, but also about promoting social and psychological well-being as well. The ultimate goal of TBI rehabilitation is to help individuals reintegrate into society and become active members of their community (51). Social support is a key factor facilitating community integration, while physical environment and fatigue are often identified as barriers (52). Although the CIQ, specifically developed for the TBI population, is currently the standardized measure of CI outcomes (53), more work is needed to develop inclusive, culturally sensitive, and appropriate tools (54, 55).

In the aspect of TBI management, research primarily addresses concerns in physical therapy and cognitive and psychological management. Inadequate management of mild TBI may place patients at risk for second impact syndrome and chronic traumatic encephalopathy. Quatman-Yates et al. (45) proposed a novel clinical guideline focused on optimizing physical therapy management for mild TBI. The emerging “active rehabilitation” paradigm, emphasizing active interventions and specialized rehabilitation techniques, has rendered physical therapists vital in interdisciplinary care for individuals with mild TBI (56, 57). The emerging consensus underscore the necessity of tailoring cognitive rehabilitation according to individuals’ unique profiles, goals, and pre-injury activities, employing diverse approaches such as group therapy to promote generalization and concentrate on personally meaningful activities within the individuals’ environment (43, 58). Fenton et al. (59) reported that 39% of individuals with TBI were diagnosed with a psychiatric condition 6 weeks post injury. Cognitive behavioral therapy is the preferred therapeutic approach for treating mental disturbances, with related therapies like dialectical behavior, mindfulness, and acceptance and commitment therapies being proposed (60, 61). Further research is required to validate the efficacy of these approaches.

Emerging research hotspots in TBI rehabilitation include “injury chronicity” and “sequelae.” Recent research has shifted focus toward long-term effects of TBI on individuals, including chronic neurobehavioral sequelae such as cognitive dysfunction, personality changes, and increased rates of psychiatric illness (62, 63). Evidence is mounting that TBI can have an impact on an individual’s health and function years after onset (64). In fact, greater injury chronicity is associated with higher levels of disability, reduced functional independence, and lower levels of community participation (65). Other frequently occurring keywords in the literature include “mild TBI,” “children,” and “stroke.” Mild TBI, also known as concussion, represents between 75 and 90% of all TBIs (66) and often lacks adequate follow-up care. Although most people who experience mild TBI fully recover within a few weeks, research indicates that up to 15% of patients diagnosed with mild TBI may experience persistent, disabling problems (67). Children are a crucial population in TBI rehabilitation research, as TBI can affect them differently from adults. In children, some health effects, such as deficits in organization and problem-solving, may be delayed and not surface until later (68). Stroke is also the most commonly occurring keyword in the rehabilitation of cardiovascular and cerebrovascular diseases field (69). Furthermore, it is the most frequently studied neurological disease, with 39% of the 100 most cited papers on neurorehabilitation focusing on stroke (70). An overlay visualization map of the keyword co-occurrence revealed research frontiers in TBI rehabilitation, including “return to work,” “disorder of consciousness,” “veterans,” “mild TBI,” “pediatric,” “executive function,” and “acquired brain injury.” These areas also focus on CI and TBI management, which represent crucial directions for future research.

A limitation of this study is that it did not include PubMed and Scopus databases. This decision was made because bibliometric analysis using PubMed does not allow for citation and co-citation analysis, while the Scopus database has a low impact level and is not indexed in some journals (71). WoS is considered a more reliable database due to its indexing of high-impact journals. Furthermore, studies analyzing a large number of articles may encounter the issue of duplicate inclusion when utilizing multiple databases. Despite this limitation, this study’s strength and superiority lie in its comprehensive bibliometric analysis, which is unmatched by other studies in the literature.

## 5. Conclusion

This study systematically summarized the articles on TBI rehabilitation research from 1988 to 2022 through bibliometric analysis using visualization tools. The results highlighted increasing attention and interest in TBI rehabilitation research, characterized by a multidisciplinary approach. The US, Australia, and Canada were identified as leaders in TBI rehabilitation research, with the University of Washington playing a central role in collaborative research efforts. Co-citation references primarily focused on outcome assessment, CI, and TBI management, with “injury chronicity” and “sequelae” receiving particular attention in recent years. The analysis also uncovered emerging research frontiers, including “return to work,” “disorder of consciousness,” “veterans,” “mild TBI,” “pediatric,” “executive function,” and “acquired brain injury.” By examining patterns and trends in TBI rehabilitation research, this study provided valuable insights for a better understanding of the current state of research and may inform future research directions.

## Author contributions

YL: conceptualization and writing—original draft preparation. YL and XY: methodology, data curation, and investigation. YL, XY, and JQ: writing—review and editing. JQ: supervision, funding acquisition, and project administration. All authors contributed to the article and approved the submitted version.

## Funding

This research was supported by a grant from the Beijing High-Grade, Precision, and Advanced Disciplines Project.

## Conflict of interest

The authors declare that the research was conducted in the absence of any commercial or financial relationships that could be construed as a potential conflict of interest.

## Publisher’s note

All claims expressed in this article are solely those of the authors and do not necessarily represent those of their affiliated organizations, or those of the publisher, the editors and the reviewers. Any product that may be evaluated in this article, or claim that may be made by its manufacturer, is not guaranteed or endorsed by the publisher.
