# Supplementary material for: Thirty years of research on traumatic brain injury rehabilitation: a bibliometric study
Source: Front Neurol. 2023 May 15;14:1170731. doi: 10.3389/fneur.2023.1170731 (PMC10225562; doi:10.3389/fneur.2023.1170731)
Supplement: Supplementary file 2 [file Data_Sheet_2.docx]

Supplementary Material

Thirty years of research on Traumatic Brain Injury Rehabilitation: A Bibliometric Study

Yang Liu, Xiaomeng Yao, Jinghua Qian^*^

*** Correspondence:** Jinghua Qian: [JinghuaQian891273@126.com](mailto:JinghuaQian891273@126.com)

# Supplementary Figure


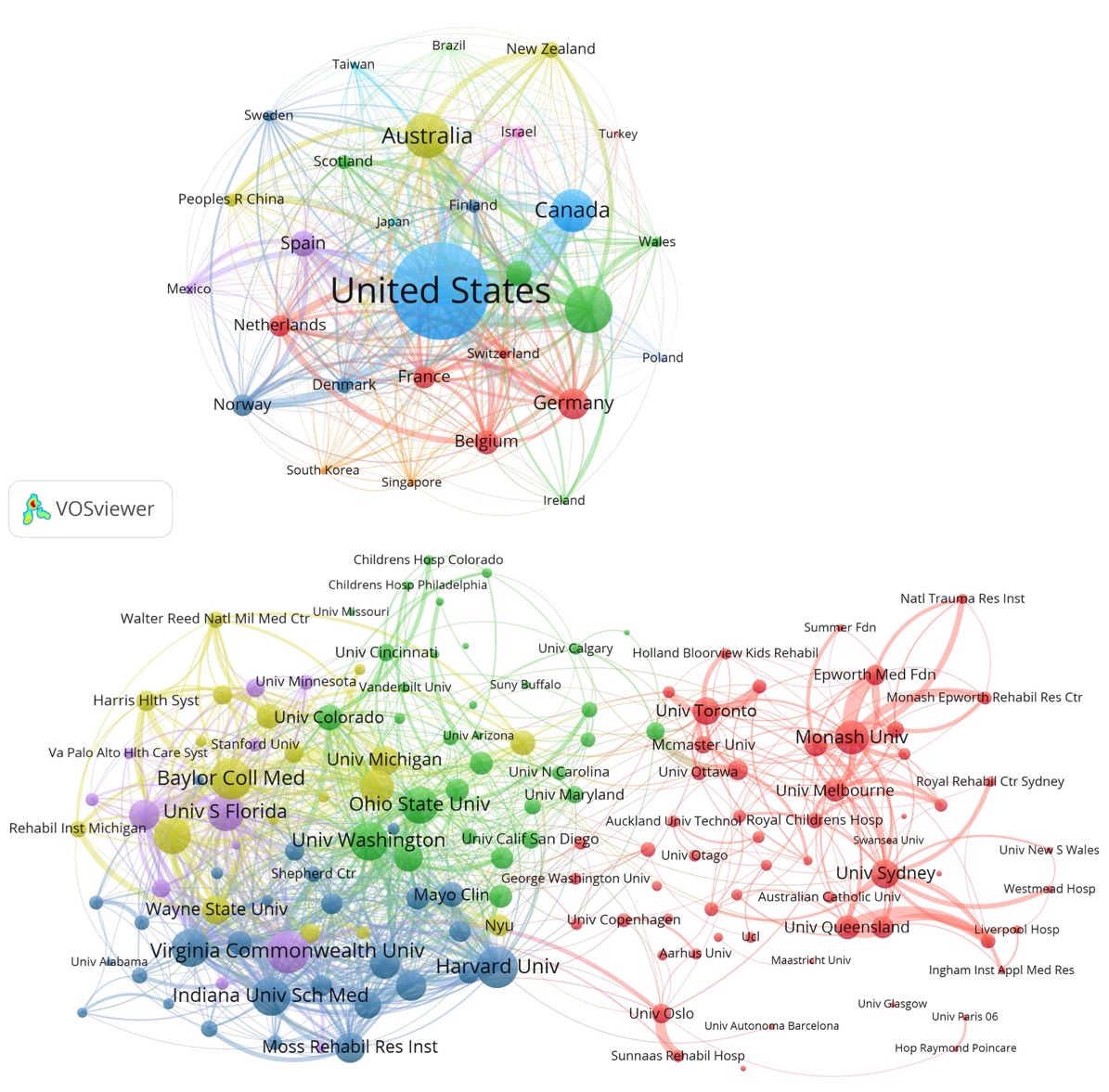


A

B

Supplementary Figure 1 Collaboration network map of countries/regions (A) and institutions (B)in TBI rehabilitation research.


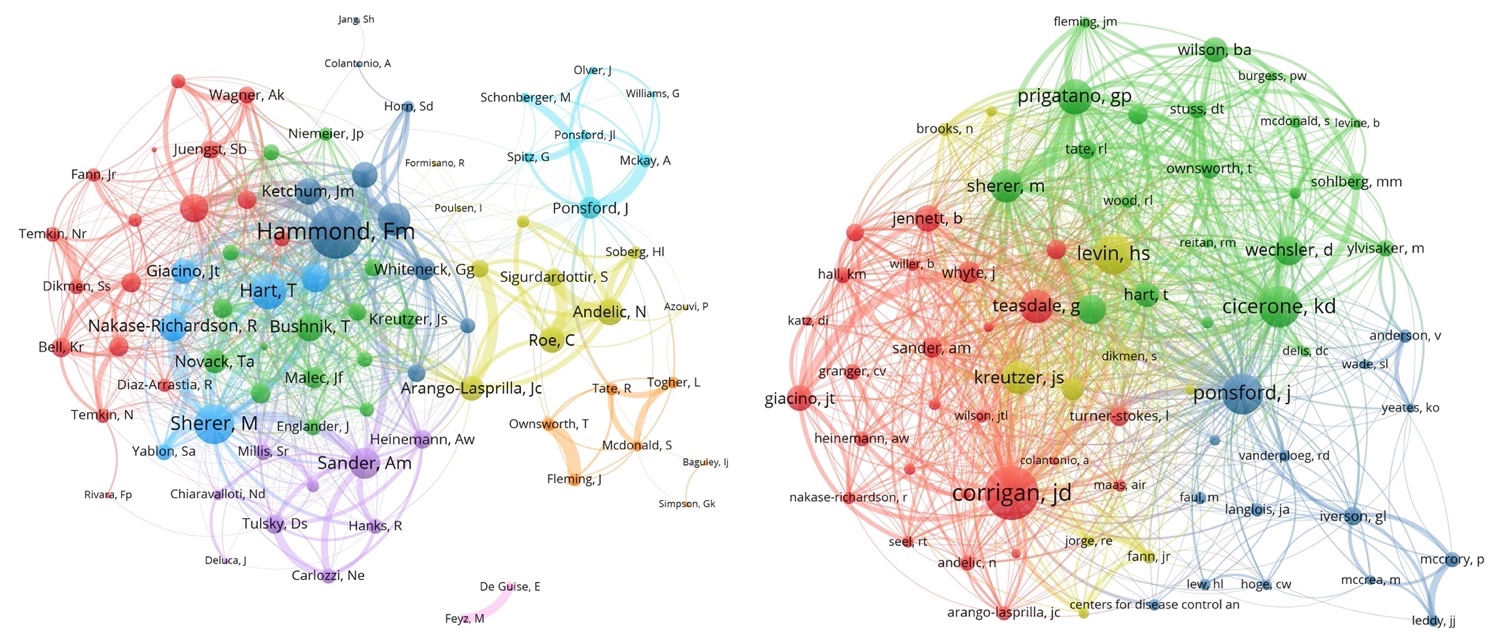


B

A

Supplementary Figure 2 Collaboration network map (A) and co-citation network map (B) of authors in TBI rehabilitation research.


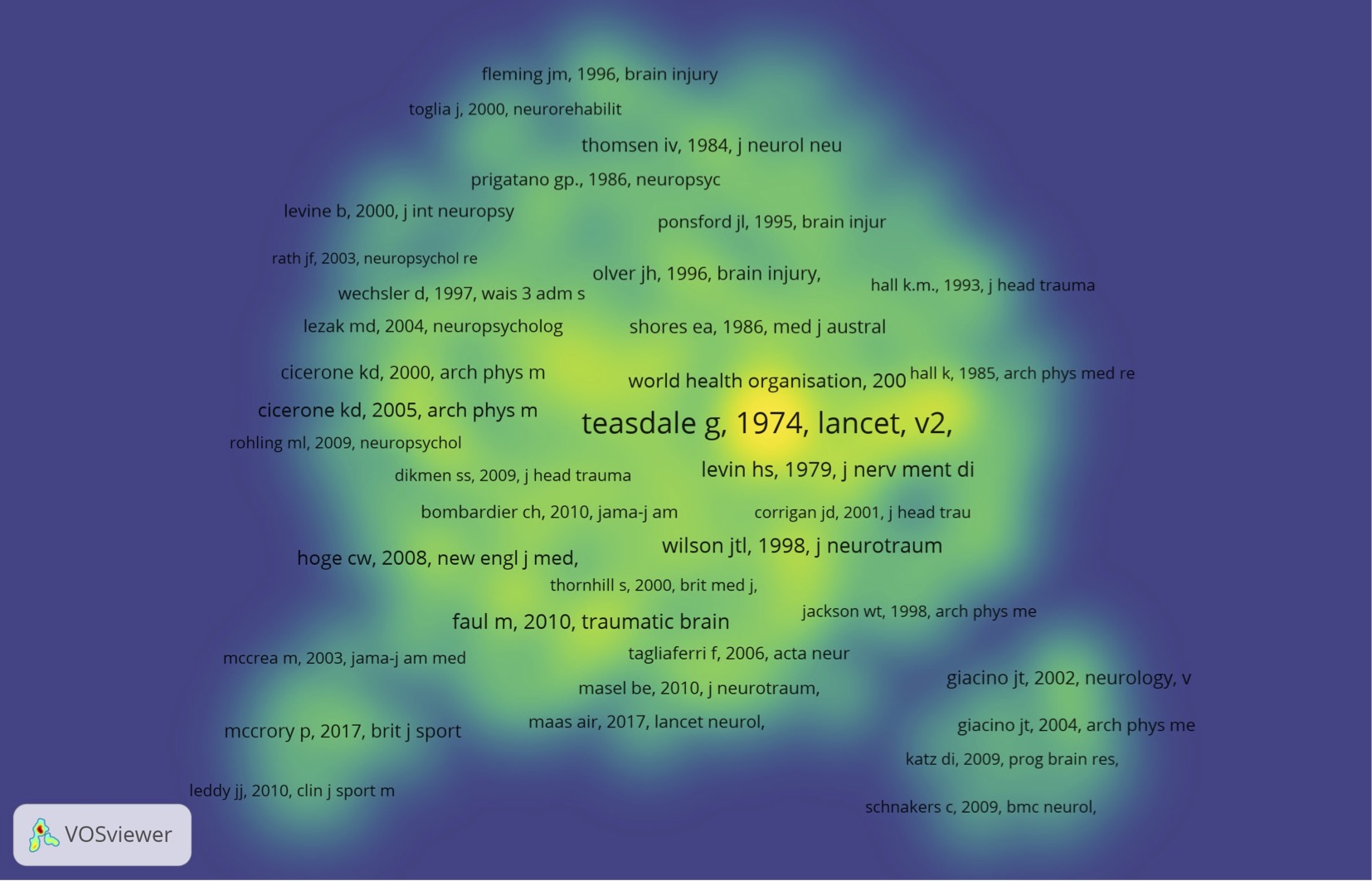


Supplementary Figure 3 Density visualization map for co-citation analysis in references on TBI Rehabilitation. (Colors in the figure range from blue to green to yellow. Points with a higher density of neighboring items and greater weights are closer to yellow, while points with fewer neighbors and lower weights are closer to blue.)


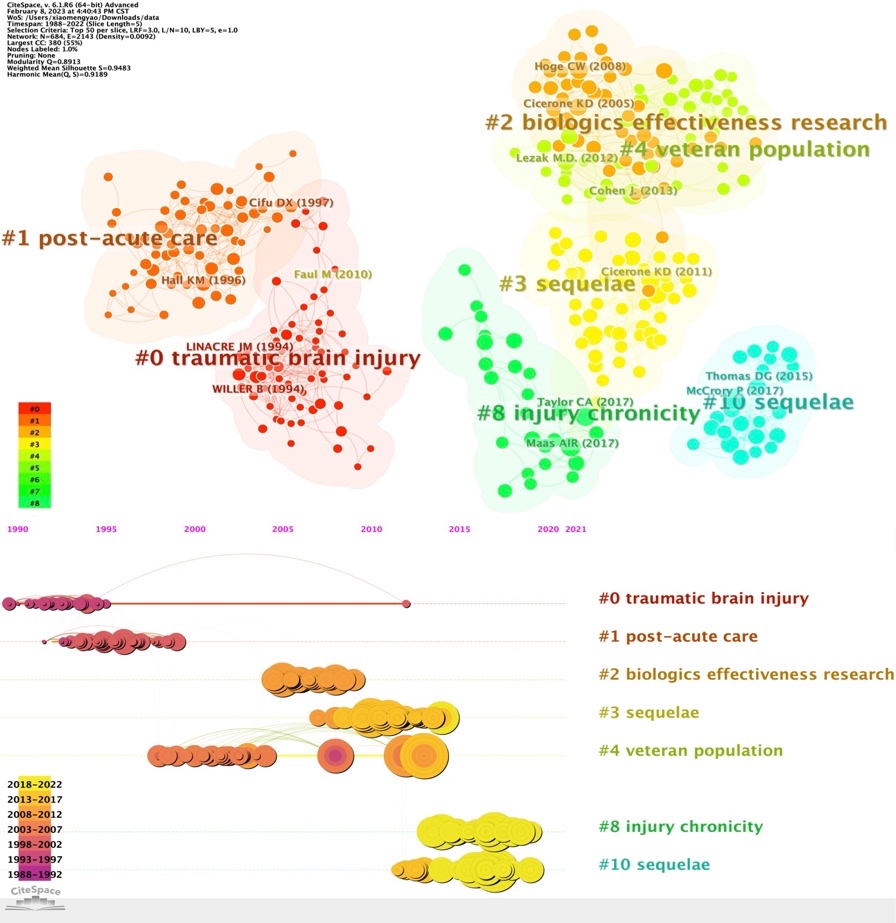


A

B

Supplementary Figure 4 Cluster network map (A) and cluster timeline map (B) of reference co-citation on TBI Rehabilitation.


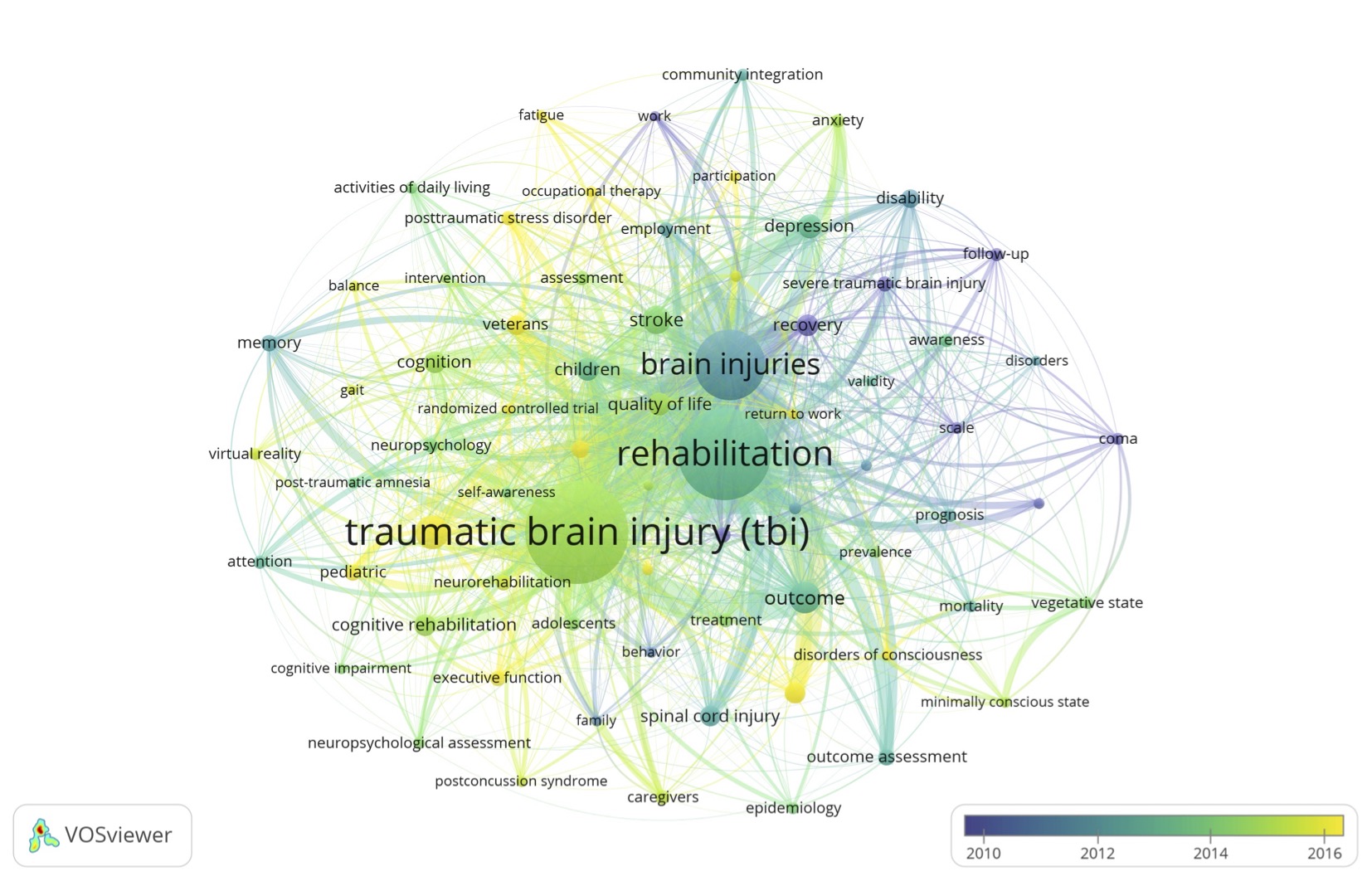


Supplementary Figure 5 Overlay visualization map of keywords co-occurrence.

# Supplementary Table

Supplementary Table 1 The Top 10 most cited articles

| Article Title | First Author | PY | NC | AC |
| --- | --- | --- | --- | --- |
| Treatment of traumatic brain injury with moderate hypothermia | Marion, DW (21) | 1997 | 940 | 37.6 |
| Position Statement: Definition of Traumatic Brain Injury | Menon, DK (22) | 2010 | 827 | 63.6 |
| Global, regional, and national burden of neurological disorders, 1990-2016: a systematic analysis for the Global Burden of Disease Study 2016 | Feigin, VL (2) | 2019 | 695 | 173.8 |
| Constraint-Induced Movement Therapy: A new family of techniques with broad application to physical rehabilitation - A clinical review | Taub, E (23) | 1999 | 608 | 26.4 |
| Traumatic Brain Injury Screening: Preliminary Findings in a US Army Brigade Combat Team | Terrio, H (58) | 2009 | 586 | 41.9 |
| Traumatic Brain Injury: A Disease Process, Not an Event | Masel, BE (53) | 2010 | 575 | 44.2 |
| Reliability and validity of arm function assessment with standardized guidelines for the Fugl-Meyer Test, Action Research Arm Test and Box and Block Test: a multicentre study | Platz, T (59) | 2005 | 537 | 29.8 |
| Major Depression Following Traumatic Brain Injury | Jorge, Re (60) | 2004 | 479 | 25.2 |
| Prevalence Of Chronic Pain, Posttraumatic Stress Disorder, And Persistent Postconcussive Symptoms In Oif/Oef Veterans: Polytrauma Clinical Triad | Lew, Hl (61) | 2009 | 447 | 31.9 |
| Rates Of Major Depressive Disorder And Clinical Outcomes Following Traumatic Brain Injury | Bombardier, Ch (62) | 2010 | 438 | 33.7 |
| PY: Publication year; NC: Numbers of citations; AC: Average citations per year. | | | |  |

# Supplementary Data

Supplementary material is the data used in the bibliometric analysis of this study.
